# Uterine cervix metastasis from a sigmoid adenocarcinoma: a rare presentation of an uncommon tumor

**DOI:** 10.1186/2053-6844-1-6

**Published:** 2014-12-01

**Authors:** Soufiane Berhili, Basma El Khannoussi, Selma Kadiri, Imane Mezouri, Amine Bazine, Asmae Touil, Imane El Khiyat, Tayeb Kebdani, Noureddine Benjaafar

**Affiliations:** Department of Radiotherapy, National Institute of Oncology, Ibn Sina University Hospital, Mohamed 5 Souissi University, Rabat, Morocco; Laboratory of Cytopathology, National Institute of Oncology, Ibn Sina University Hospital, Mohamed 5 Souissi University, Rabat, Morocco

**Keywords:** Uterine cervix, Metastasis, Adenocarcinoma, Sigmoid cancer, Immunohistochemistry

## Abstract

Metastatic carcinoma to the uterine cervix from colorectal cancer, through haematogenous or lymphatic spread, is extremely rare. We report the case of a 59 year old woman in whom cervical metastasis was diagnosed after 13 months of follow-up for a sigmoid adenocarcinoma, confirmed by immunohistochemical study, with a review of the literature. This case illustrates that abnormal gynecologic symptoms can reveal a disease progression on patients who suffer colorectal cancer.

**Electronic supplementary material:**

The online version of this article (doi:10.1186/2053-6844-1-6) contains supplementary material, which is available to authorized users.

## Background

Metastases to the female genital tract from extragenital malignancies are very uncommon, and the most common extra-genital primary sites are breast and gastrointestinal system [1, 2]. Common metastatic sites of colorectal cancer include the liver, lung, lymph nodes and peritoneum, but the rarest would be the uterine cervix [3, 4]. We report in this article a case of metastatic adenocarcinoma from a sigmoid primary to the uterine cervix, with a review of literature on uterine cervix metastasis from colorectal cancer.

## Case presentation

**Figure 1:**
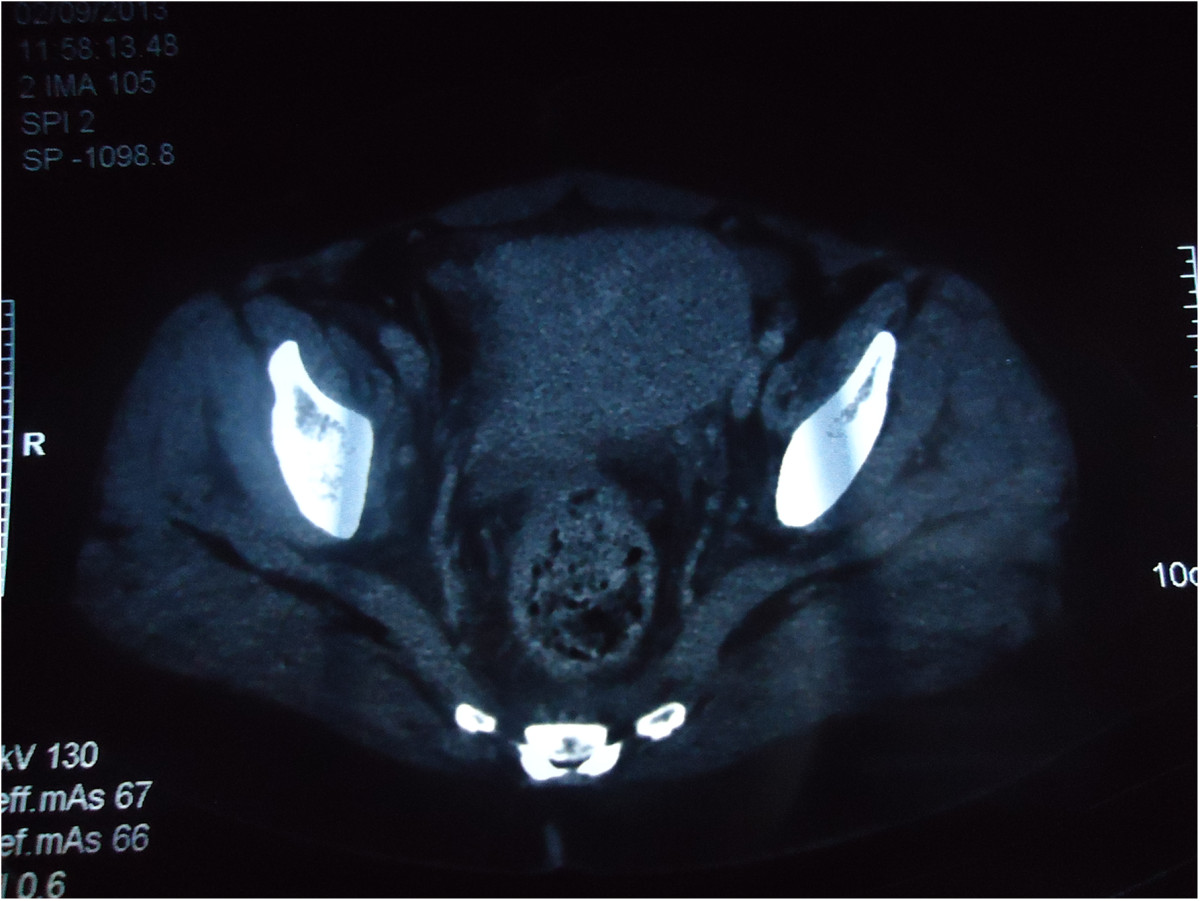
**Computed tomography slice of the patient pelvis showing a cervico-isthmic mass.**

**Figure 2:**
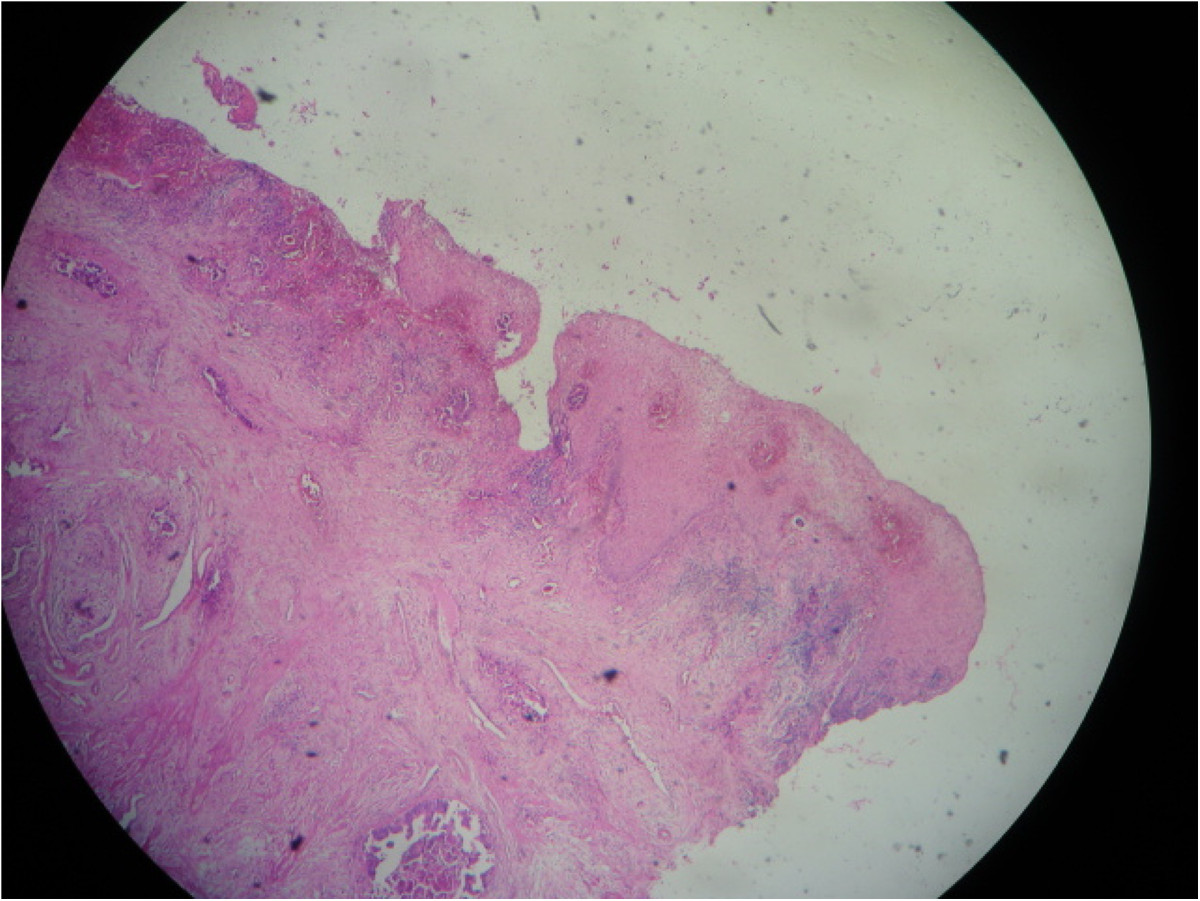
**Cervical biopsy showing metastatic moderately differentiated adenocarcinoma cells: Ectocervical mucosa infiltrated by a carcinomatous proliferation of glands of varying size and focus of necrosis lined by atypical cells.**

**Figure 3:**
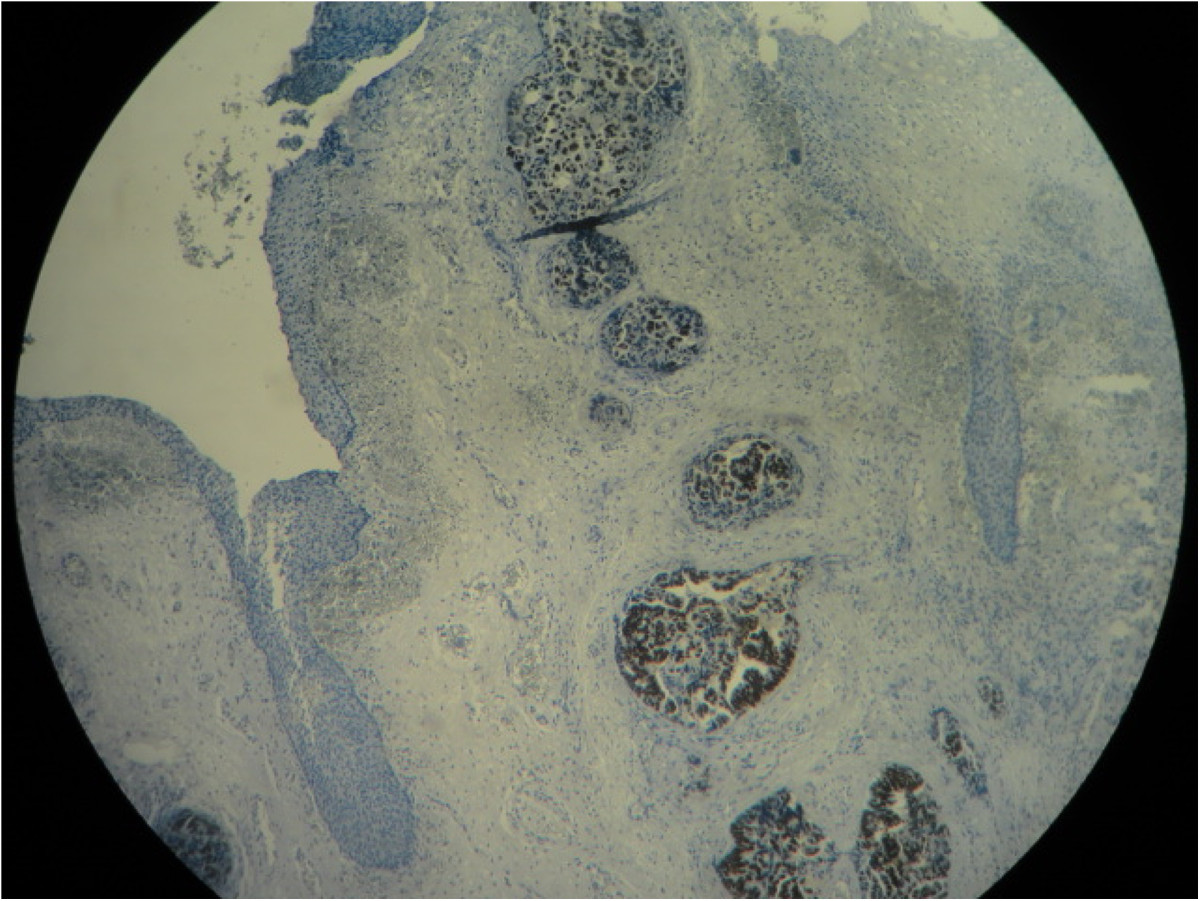
**Immunohistochemical study of cervical tumor cells: Strong nuclear staining of tumor cells by the CDX2 marker.**

A 59-year-old woman presented in our department with spinal cord compression at the fifth thoracic vertebrae (T5) and epiduritis, in order to receive radiotherapy on vertebral metastasis from a primary well-differentiated adenocarcinoma of the sigmoid that was diagnosed one year earlier. The patient was initially operated in September 2012 undergoing a laparotomic sigmoidectomy, post-operative cytopathology concluded in a pT4 N2 M0 stage with negative margins (R0 resection). She received eight cycles of adjuvant chemotherapy (Capecitabine + Oxaliplatine) after that time at the Day-hospital of the National Institute of Oncology in Rabat, without significant adverse drug effects. During this period, physical examination and computed tomography (CT) did not detect any local or distant relapse of the disease.In September 2013, the patient reported whitish vaginal discharges lasting for three months earlier. In our department, physical examination did not find any neurological deficit but rather back pain lasting for 15 days due to spinal metastasis. Gynecologic examination was painful and found an easy bleeding ulcerative cervical tumor measuring approximately 6 cm with vaginal and bilateral parametrial involvement at rectal digital examination. Thoracic-abdominal-pelvic CT showed T5 spinal cord compression with signs of epiduritis, right pleurisy, ascites, and cervical mass extended to the corpus and measuring 68 × 70 mm, without bladder or rectal invasion (Figure 1). No secondary lesions were found in lung or liver. CA 19–9 serum tumor marker rate was 821 U/ml (normal <35 U/ml).Cervical biopsy was performed and showed a moderately differentiated adenocarcinoma of colonic origin (Figure 2), confirmed by immunohistochemical study showing tumor cells diffusely and strongly positive for CK20 and CDX2 markers, and negative for CK7 marker (Figure 3).

10 daily fractions of decompression radiotherapy were scheduled (3 Gy per fraction), but the patient received only six fractions, she died before completing his treatment, two days after presenting a coma secondary to brain metastases, asymptomatic at the time of admission. Cervical tumor has therefore not been treated.

Involvement of the uterine cervix through direct extension of extra-genital neoplasia is frequent. However, metastatic carcinoma to the cervix through haematogenous or lymphatic spread is extremely rare [1, 3]. Contrary to ovaries which provide a suitable environment for metastatic cells, the uterine cervix is rarely the site of metastases because of its fibrous tissue content, small size, relatively limited blood flow, and the lymphatic vessels of the pelvis all draining away from the cervix [5]. Thus, the uterus is rarely involved accounting for less than 10% of all cases of metastases to the female genital tract from extra-genital cancers (3.4% for the uterine cervix alone) [1]. Only few cases have been reported during the past several decades; the youngest patient was 17 years old [6]. These patients carry a dismal prognosis [7].

The gastrointestinal tract tumors are the most preponderant extra-genital primaries of the uterine cervix metastasis which are the stomach, colon, rectum, breast, and skin melanoma [8, 9].

Nakagami et al. [10], reviewed 27 cervical metastases from colorectal carcinomas, including a majority of colonic primaries. The average interval between primary carcinoma and uterine cervix metastasis appearance was 17 months (0–60 months), it was 13 months for our patient. The average survival after cervical metastasis diagnosis in Nakagami’s review was 11 months.

In our case, leucorrhea appeared 3 months before the diagnosis of metastasis. Gynecological symptoms following medical history of colorectal cancer should be taken into consideration in order to refer rapidly patients for adequate treatment.

Adenocarcinoma histology type represents 0.42 to 11.7% of all cervix carcinomas [11], however the incidence of metastatic adenocarcinoma was reported to be 21.6 to 56.9% of cervical adenocarcinoma [11, 12].

In pathology study practice, CK7 and CK20 immunoexpresssions are commonly used in combination to evaluate immunoreactivity pattern in tumor cells, to confirm the origin of metastatic disease with unknown primary sites [13]. CK7 immunoreactivity is observed in tumors of lung, breast, upper gastrointestinal and pancreatobiliary tract, endometrium, vagina, and ovary, while tumors of colonic origin are generally non-reactive to this immunomarker. CK20 immunoexpression is detected in tumors of gastrointestinal tract and urothelium [14]. CDX2 immunoexpression has proven to be useful in establishing gastrointestinal origin in metastatic tumors, and has become a useful addition to the standard immunohistochemical stains list for carcinomas of unknown primary sites [15].

Thereby, in routine clinical practice and as we did, a panel combining [CK7-/CK20+/CDX2+] is used to confirm colonic primary origin [9, 13].

In our case, the spread of the tumor to the uterine cervix might have been caused by lymphatic and/or hematogenous pathways. Since lesion by direct extension from the primary sigmoid tumor was excluded (R0 resection was performed), and lymphatic channels of the cervix that routinely drain centrifugally, were blocked by surgery and retrograde flow might occur [5, 16, 10].

## Conclusions

In conclusion, metastases to the female genital tract from extragenital cancers are uncommon and have a poor prognosis [9, 10]. Multidisciplinary approach including immunohistochemical study is required to diagnose these rare cases, and any gynecological symptoms following medical history of colorectal carcinoma should bear in mind metastases in order to appropriately assist these patients.

## Consent

Written informed consent was obtained from the next of kin of the patient for publication of this Case report and any accompanying images. A copy of the written consent is available for review by the Editor-in-Chief of this journal.

## Authors’ original submitted files for images

Below are the links to the authors’ original submitted files for images. Authors’ original file for figure 1 Authors’ original file for figure 2 Authors’ original file for figure 3 Authors’ original file for figure 4 Authors’ original file for figure 5 Authors’ original file for figure 6
