# Supplementary figures and images for: Uterine cervix metastasis from a sigmoid adenocarcinoma: a rare presentation of an uncommon tumor
Source: Gynecol Oncol Res Pract. 2014 Dec 1;1:6. doi: 10.1186/2053-6844-1-6 (PMC4878055; doi:10.1186/2053-6844-1-6)

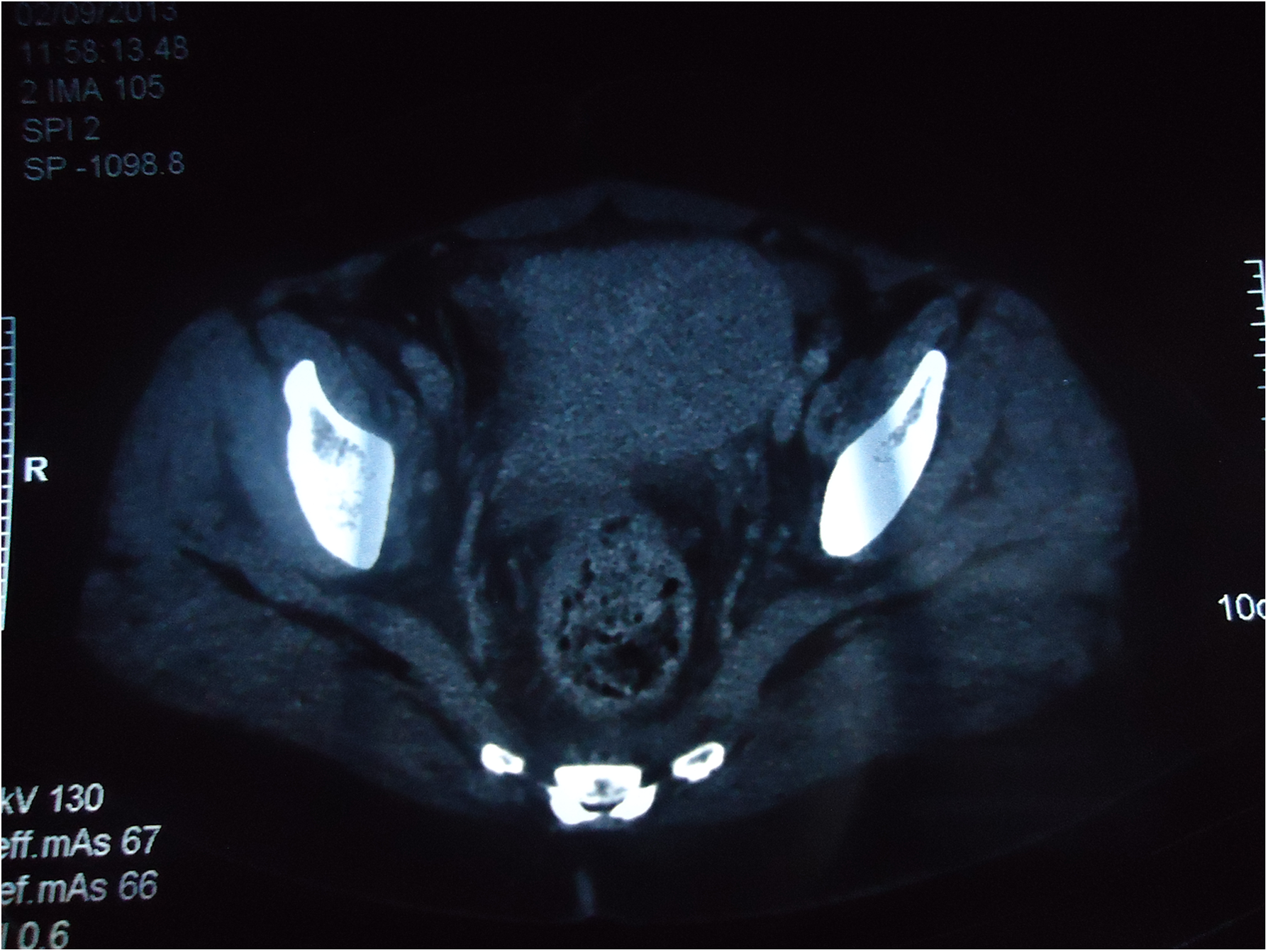

Supplement: Supplementary file 1 — Authors’ original file for figure 1 [file 40661_2014_4_MOESM1_ESM.tiff]

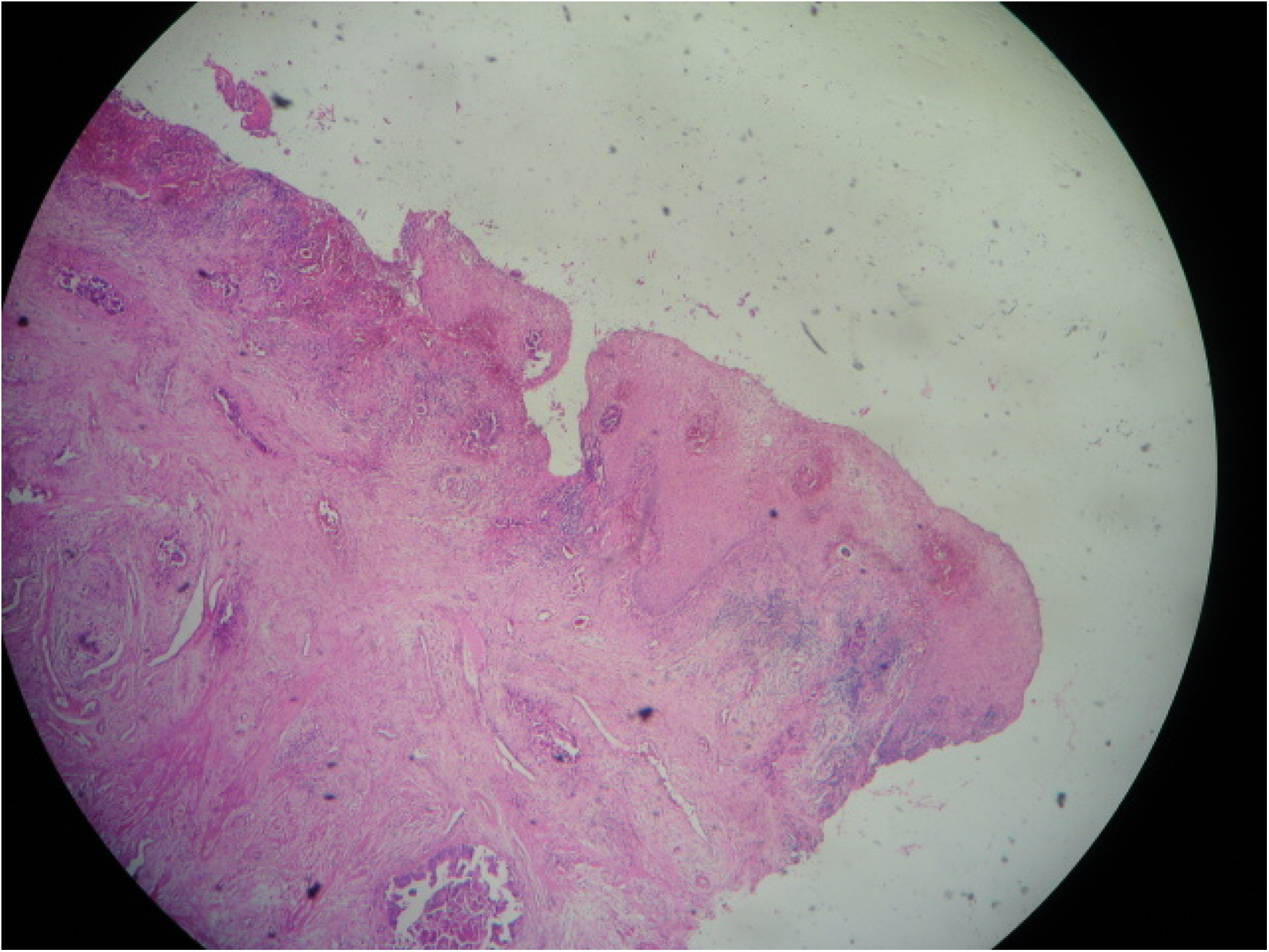

Supplement: Supplementary file 2 — Authors’ original file for figure 2 [file 40661_2014_4_MOESM2_ESM.tiff]

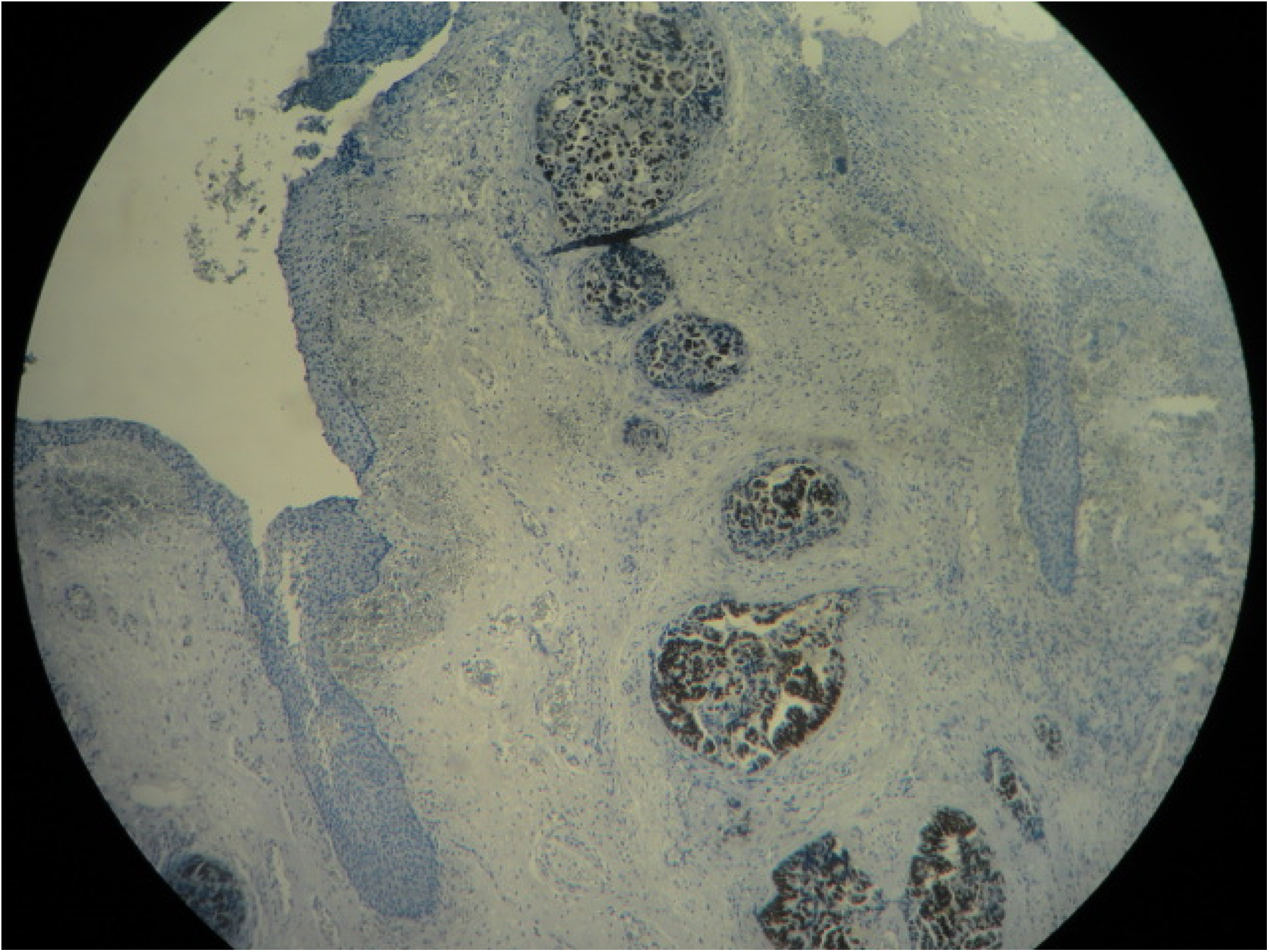

Supplement: Supplementary file 3 — Authors’ original file for figure 3 [file 40661_2014_4_MOESM3_ESM.tiff]
